# Percutaneous coronary intervention versus coronary artery bypass grafting in left main disease according to patients' sex: A meta‐analysis

**DOI:** 10.1111/eci.14348

**Published:** 2024-11-14

**Authors:** Pierre Meynet, Riccardo Improta, Maria Luisa Carbone, Martina Pecoraro, Ilaria Pagliassotto, Gianluca Di Pietro, Michelle Demetres, Francesco Bruno, Gaia Comitini, Attilio Leone, Eleonora Martinengo, Stefano Siliano, Fabrizio D'Ascenzo, Alaide Chieffo, Gaetano Maria De Ferrari, Mario Gaudino, Massimo Mancone, Antonino Di Franco, Ovidio De Filippo

**Affiliations:** ^1^ Division of Cardiology, Cardiovascular and Thoracic Department "Città della Salute e della Scienza" Hospital Turin Italy; ^2^ Department of Medical Sciences University of Turin Turin Italy; ^3^ Department of Clinical Internal, Anaesthesiological and Cardiovascular Sciences Sapienza University of Rome Rome Italy; ^4^ Samuel J. Wood Library and C.V. Starr Biomedical Information Center Weill Cornell Medicine New York New York USA; ^5^ Division of Cardiology SG Moscati Hospital Avellino Italy; ^6^ Department of Advanced Biomedical Sciences University of Naples Federico II Naples Italy; ^7^ Interventional Cardiology Unit IRCCS San Raffaele Scientific Institute Milan Italy; ^8^ Vita Salute San Raffaele University Milan Italy; ^9^ Department of Cardiothoracic Surgery Weill Cornell Medicine New York New York USA

**Keywords:** coronary artery bypass graft, female, male, percutaneous coronary intervention, sex, unprotected left main

## Abstract

**Background:**

The role of sex in choosing between coronary artery bypass grafting (CABG) and percutaneous coronary intervention (PCI) for unprotected left main coronary artery (ULMCA) disease has gained interest.

**Methods:**

Randomized controlled trials and adjusted observational studies comparing PCI versus CABG in ULMCA patients with outcomes by sex were included. The primary endpoint was major adverse cardiovascular events (MACE), with secondary endpoints being all‐cause mortality and repeated revascularization.

**Results:**

Ten studies (3 randomized, 7 observational) involving 22,141 ULMCA disease patients (13,411 PCI, 8730 CABG) with a median 5‐year follow‐up were included. Among males, PCI was associated with a higher risk of MACE (HR 1.18, 95% CI 1.01–1.38), while no significant difference was seen in females. However, moderator analysis showed no significant interaction between sex and revascularization strategy for MACE (*p* for interaction .422). No differences in all‐cause mortality were observed between PCI and CABG for either sex. Repeated revascularization risk was significantly higher with PCI for both sexes (HR 3.51, 95% CI 2.21–5.59 in males and HR 4.20, 95% CI 2.57–6.87 in females).

**Conclusions:**

In males with ULMCA disease, CABG was associated with a lower risk of MACE compared to PCI, while no significant differences were seen in females. The lack of a significant interaction between sex and revascularization strategy suggests that these findings may not reflect true sex‐based effect modification. PCI was linked to a higher risk of repeated revascularization in both sexes compared to CABG.

**Trial registration:**

The protocol was registered in the PROSPERO International Prospective Register of Systematic Reviews (ID: CRD42024537726).

## Graphical abstract

Among male patients with unprotected left main coronary artery disease, coronary artery bypass grafting was associated with a significantly lower risk of major adverse cardiovascular events (MACE). However, moderator analysis showed no significant interaction between sex and revascularization strategy for MACE. No differences between percutaneous coronary intervention and coronary artery bypass grafting were observed in females. In both male and female patients, percutaneous coronary intervention was associated with a higher risk of repeated revascularization compared with coronary artery bypass grafting.

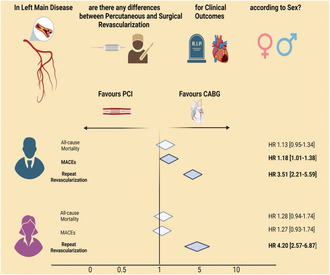

## 1 INTRODUCTION

Coronary revascularization stands as the cornerstone of treatment for unprotected left main coronary artery (ULMCA) disease.^1^, ^2^ While coronary artery bypass grafting (CABG) has traditionally demonstrated superior long‐term outcomes, evidence has emerged supporting percutaneous coronary intervention (PCI) as a viable alternative, yielding non‐inferior outcomes across various contexts and patient cohorts.^3^, ^4^ Current guidelines advocate for a tailored approach, considering coronary artery disease (CAD) complexity when choosing between PCI and CABG, with a state of equipoise particularly noted for patients presenting with low SYNTAX scores.^5^ Nonetheless, beyond SYNTAX scores, an array of variables may exert influence on revascularization outcomes. Among these, the role of patients' sex has gained significant interest, prompting numerous post‐hoc analyses to explore its impact on treatment efficacy.

While previous studies have suggested poorer outcomes for females undergoing PCI, such disparities have often been ascribed to differences in cardiovascular risk profiles between sexes, with some attenuation observed upon adjustment for confounding factors.^6^, ^7^ However, in the specific context of ULMCA disease, evidence remains inconclusive. Notably, in the EXCEL (Evaluation of XIENCE versus Coronary Artery Bypass Surgery for Effectiveness of Left Main Revascularization) trial, a non‐significant trend towards a higher occurrence of the primary composite endpoint compared to CABG at 3 years was reported for female patients treated with PCI, despite presenting with significantly lower SYNTAX scores compared to males.^8^ Similarly, the SYNTAX (Synergy Between Percutaneous Coronary Intervention With Taxus and Cardiac Surgery) trial, including patients with multivessel and ULMCA disease, reported higher 4‐year mortality rates among females undergoing PCI, whereas CABG outcomes did not show sex‐based disparities.^9^ These results were further corroborated by observations from the DELTA registries.^10^

Conversely, a post‐hoc analysis of the PRECOMBAT (Bypass Surgery Versus Angioplasty Using Sirolimus‐Eluting Stent in Patients With Left Main Coronary Artery Disease) trial did not identify inferior outcomes in females undergoing PCI for ULMCA disease.^11^

Given the discordance within existing literature, our objective was to conduct a comprehensive meta‐analysis encompassing randomized controlled trials (RCTs) and adjusted observational studies to systematically compare the outcomes of CABG versus PCI in ULMCA disease stratified according to patients' sex, with the aim of elucidating the interplay between treatment modality and sex‐specific outcomes.

## 2 METHODS

The present analysis was conducted in accordance with the PRISMA (Preferred Reporting Items for Systematic Reviews and Meta‐Analyses) guidelines^12^ and was pre‐registered in the international prospective register of systematic reviews (PROSPERO; ID: 537726).

### 2.1 Search strategy

A medical librarian (MD) performed comprehensive searches to identify all RCTs and adjusted observational studies that compared PCI versus CABG for the treatment of ULMCA disease. Searches were run on January 31, 2024 in the following databases: Ovid MEDLINE®, Ovid EMBASE and The Cochrane Library (Wiley). The search strategy included the following terms: ‘percutaneous coronary intervention’, ‘PCI’, ‘stent’, ‘angioplasty’, ‘percutaneous transluminal coronary angioplasty’, ‘PTCA’, ‘coronary artery bypass’, ‘CABG’, ‘off pump coronary artery bypass’, ‘left internal mammary artery’, ‘left internal thoracic artery’, ‘myocardial revascularization’, ‘left main’ (details about the full search strategy are available in the Appendix S2).

### 2.2 Study selection and data extraction

Database searches were de‐duplicated by a qualified medical librarian. Three investigators (PM, MP and MLC) screened the searched database for inclusion and performed data extraction independently. Disagreements were resolved by a fourth author, who also checked the extracted data for accuracy (ODF).

Studies were considered for inclusion in the quantitative analysis if they were RCTs or adjusted observational studies written in English comparing PCI versus CABG for the treatment of ULMCA disease and reporting outcomes stratified according to sex category. For each included study, the publication with the longest follow‐up duration was selected. Studies reporting only in‐hospital outcomes and studies where only bare metal stents (BMS) were used were excluded. Animal studies, case reports, conference presentations, editorials, expert opinions, and non‐adjusted observational studies were also excluded. Full text for the selected studies was pulled for a second round of eligibility screening. Reference lists of articles were also searched to identify other relevant trials. Relevant baseline and procedural variables for each of the included studies were extracted for both the PCI and CABG arms. The quality of the included papers was assessed using the Cochrane Collaboration's tool for RCTs^13^ and the Newcastle Ottawa Scale^14^ for adjusted observational studies by two independent authors (RI, GC) with disagreements resolved by consensus or after consultation with a third author (ODF). Publication bias was assessed by means of Funnel plots.

### 2.3 Outcomes

A composite of all‐cause mortality, myocardial infarction (MI), stroke and repeated revascularization as defined in the individual trials (major adverse cardiovascular events, MACE) was the primary endpoint of interest. Secondary outcomes included all‐cause mortality and repeated revascularization at the longest available follow‐up.

### 2.4 Statistical analysis

Continuous variables are reported as mean (standard deviation) or median (first and third quartile). Categorical variables are expressed as *n* (%). Statistical pooling for incidence estimates was performed using random‐effect model with generic inverse‐variance weighting, computing risk estimates with 95% confidence intervals (CIs), using RevMan 5.2 (The Cochrane Collaboration, The Nordic Cochrane Centre, Copenhagen, Denmark). Hypothesis testing for superiority was set at the two‐tailed 0.05 level. We used the *I* ^2^ statistic to assess heterogeneity. Low heterogeneity was defined as 0%–25%; moderate heterogeneity was defined as 25%–50%; and substantial heterogeneity was defined as >50%. Sensitivity analysis was performed for the primary endpoint if heterogeneity resulted significantly high. An additional sensitivity analysis was performed for the primary endpoint after appraising the included studies separately according to their design (namely RCT vs. observational). Lastly, a moderator analysis using JAMOVI was performed to formally test whether sex modifies the effect of PCI versus CABG on outcomes.

## 3 RESULTS

**FIGURE 1:**
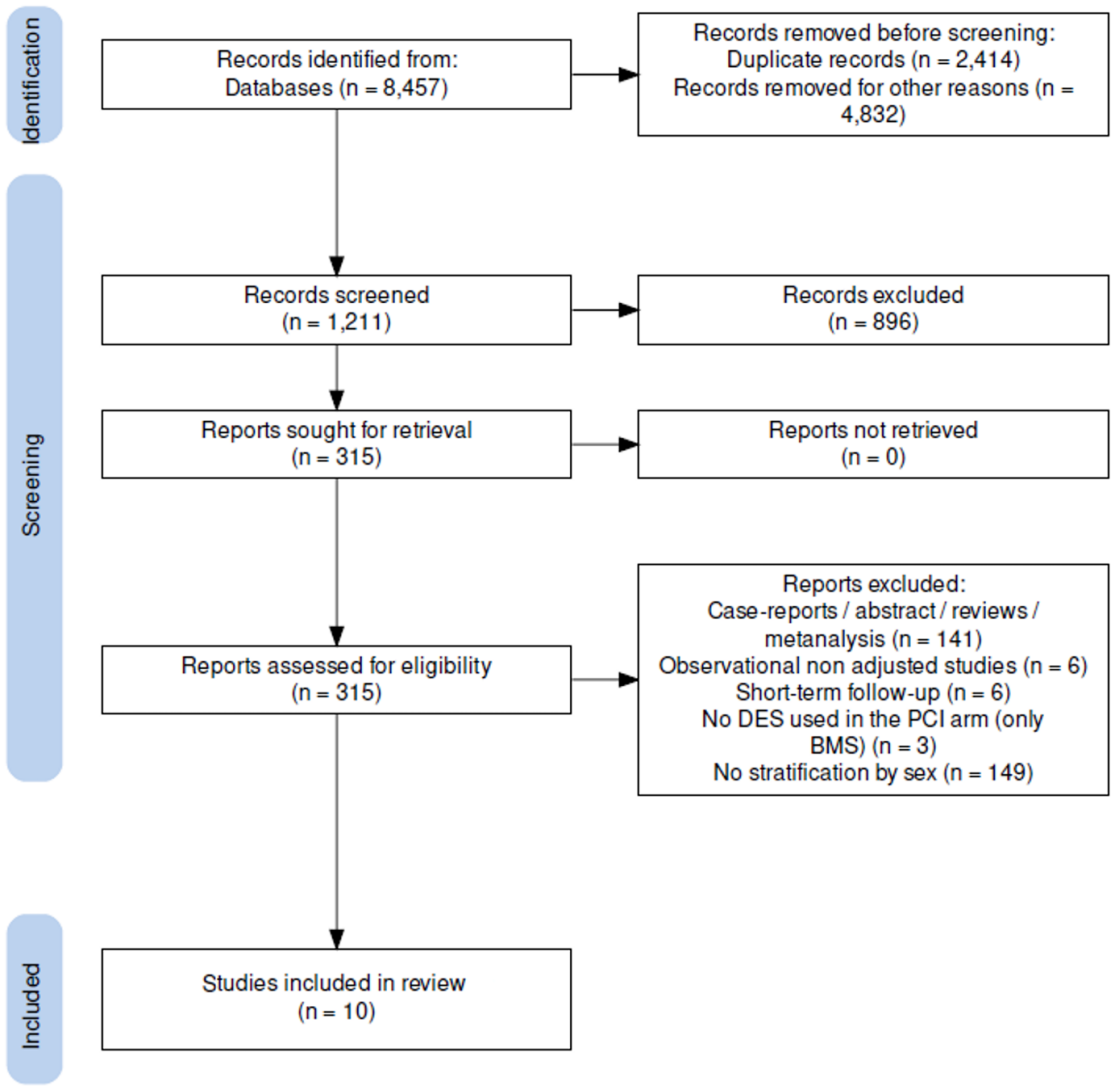
PRISMA flowchart. BMS, Bare‐metal stent; DES, Drug‐eluting stent; PCI, Percutaneous coronary intervention.

After searching for studies reporting outcomes stratified by sex, 3 RCTs^15^, ^16^, ^17^ and 7 adjusted observational studies^10^, ^18^, ^19^, ^20^, ^21^, ^22^, ^23^ were included for the quantitative analysis, globally encompassing 22,141 patients undergoing revascularization for ULMCA disease (see PRISMA flow chart, Figure 1). Overall, PCI was performed in 13,411 patients and CABG in 8730; among these, 16,879 were male and 5262 were female. Median follow‐up was 5 years (IQR 3–8.75). Characteristics of the included studies are summarized in Table 1 and Table S1 while baseline and procedural features of included patients are summarized in Tables S2–S4. Median age was 63 (IQR 62–64) and 64 years (IQR 62–66) for the male and female patients, respectively. Overall, 50% (IQR 24%–62%) of patients presented with acute coronary syndrome (ACS). Available data showed a moderate burden of diabetic patients with a median prevalence of 32% (IQR 29%–36%), with 11% (IQR 3.5%–13.5%) of patients suffering from chronic kidney disease. Regarding PCI, 66% (IQR 65%–73%) of patients were treated with a single stent technique, and intravascular imaging was used in 74% (IQR 38%–76%) of procedures. As for CABG, left internal mammal artery was used in 94% (IQR 93%–96%) of cases, with off‐pump surgery performed in 55% (IQR 32%–60%) of patients. The risk of bias assessment for observational studies and RCTs is shown in Table S5 and Figure S1, respectively. Definitions of studies' outcomes are reported in Table S6. Primary end‐point funnel plot for publication bias evaluation is displayed in Figure S2 while the results of the main analysis are presented in Table 2.

**TABLE 1:** Study characteristics.

Study	Publication year	Study design	Follow up duration (months)	Total patients (*N*)	Males (*N*)	Females (*N*)
Kang et al.^18^	2010	Observational PS adjusted	36	462	334	128
Zheng et al.^19^	2016	Observational MV adjusted	36	4046	3269	777
Hu et al.^23^	2017	Observational PS adjusted	12	276	236	40
EXCEL^15^	2019	RCT	60	1905	1464	441
NOBLE 5‐year^17^	2020	RCT	60	1184	928	256
CREDO‐Kyoto PCI/CABG Registry Cohort‐3^20^	2021	Observational MV adjusted	60	855	664	191
DELTA registries^10^	2022	Observational MV adjusted	29	6253	4564	1689
IRIS MAIN registry^21^	2022	Observational MV adjusted	120	4320	3352	968
PRECOMBAT extended^16^	2022	RCT	120	600	459	141
Main compare extended^22^	2023	Observational PS adjusted	120	2240	1609	631

Abbreviations: MV, Multivariable; N, Number; PS, Propensity Score; RCT, randomized clinical trials.

**TABLE 2:** Summary of the results of the meta‐analysis.

	Male patients		Female patients	
	Number of included studies	HR [95% CI] for PCI vs. CABG	Number of included studies	HR [95% CI] for PCI vs. CABG
MACE	9 (3 RCT + 6 observational)	**1.18 [1.01–1.38]**	9 (3 RCT + 6 observational)	1.27 [0.93–1.74]
All‐cause mortality	6 (1 RCT + 5 observational)	1.13 [0.95–1.34]	6 (1 RCT + 5 observational)	1.28 [0.94–1.74]
Repeated revascularization	4 (1 RCT + 3 observational)	**3.51 [2.21–5.59]**	4 (1 RCT + 3 observational)	**4.2 [2.57–6.87]**

*Note:* Bold values indicate statistically significant results.

Abbreviations: CI, confidence intervals; CABG, coronary artery bypass graft; MACE, major adverse cardiovascular events; HR, hazard ratio; PCI, percutaneous coronary intervention; RCT, randomized clinical trials.

### 3.1 MACE

**FIGURE 2:**
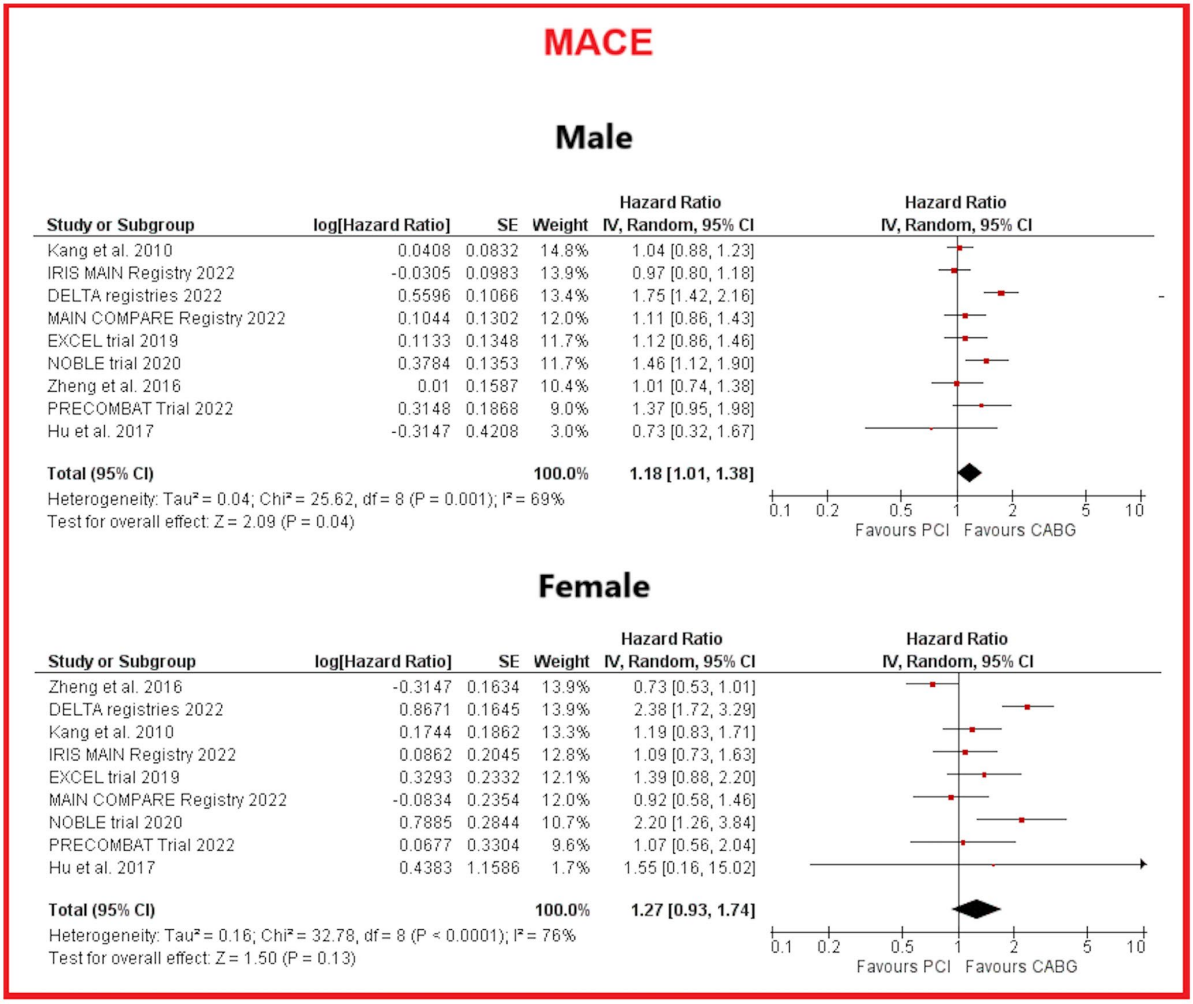
Forest plots for showing comparative outcomes for PCI versus CABG MACE in male (above) and female patients (below). CABG, coronary artery bypass graft; CI, confidence intervals; MACE, major adverse cardiovascular events; PCI, percutaneous coronary intervention; SE: standard error.

Pooled results from 9 studies (3 RCT and 6 observational), including 2768 male patients (PCI: 1836; CABG: 932) yielded a significantly higher risk of MACE in the PCI group (HR 1.18, 95% CI 1.01–1.38, *p* = .04). Among 895 female patients (PCI: 622; CABG: 273) from 9 studies (3 RCT and 6 observational), PCI and CABG did not differ for risk of MACE (HR 1.27, 95% CI .93–1.74, *p* = .13) (See Figure 2). High heterogeneity was found for both groups. A moderator analysis using sex as an interaction variable to examine the clinical impact of sex on MACE showed no significant interaction between sex and revascularization strategy (*p* for interaction .422). See Figure S3.

### 3.2 All‐cause mortality

**FIGURE 3:**
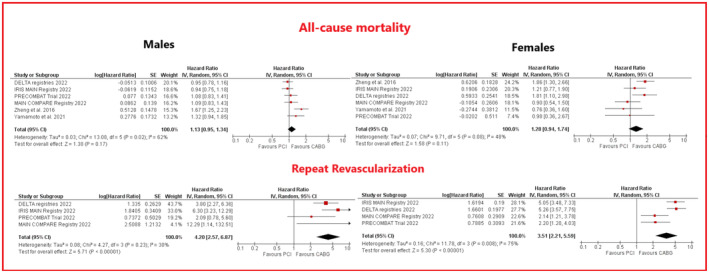
Forest plots for showing comparative outcomes for PCI versus CABG for all‐cause death in male (above) and repeat revascularization (below) in male (left panels) and female patients (right panels). CABG, coronary artery bypass graft; CI, confidence intervals; MACE: major adverse cardiovascular events; PCI, percutaneous coronary intervention; SE: standard error.

Pooled data from 6 studies (1 RCT and 5 observational) comparing PCI versus CABG among 1731 male patients showed a similar risk of all‐cause mortality (HR 1.13, 95% CI .95–1.34, *p* = .17). Also in the female group, PCI and CABG yielded similar risk of all‐cause mortality (HR 1.28, 95% CI .94–1.74, *p* = .11) (data from 6 studies, 5 observational, 1 RCT, globally including 785 patients) (see Figure 3). Heterogeneity was moderate among studies involving female patients (*I* ^2^ = 48%) while high heterogeneity was documented among studies referring to male patients (*I* ^2^ = 62%).

### 3.3 Repeated revascularization

PCI was associated with a higher rate of repeated revascularizations compared to CABG in both groups (HR 3.51, 95% CI 2.21–5.59, *p* < .001, *I* ^2^ = 75% and HR 4.20, 95% CI 2.57–6.87, *p* = <.001, *I* ^2^ = 30% for males and females, respectively) (see Figure 3).

### 3.4 Sensitivity analysis

To address the significant heterogeneity for the primary endpoint, a sensitivity analysis was performed. After inspection of funnel plots (Figure S2), outlier studies were excluded in a stepwise fashion to lower heterogeneity. After the exclusion of one study for males and two studies for females, *I* ^2^ testing resulted negative for significant heterogeneity. Sensitivity analysis results are plotted in Figure S4. For females the results were consistent with the primary analysis (HR 1.02, 95% CI .84–1.22, *p* = .87, *I* ^2^ = 15%) while for males no differences were found for revascularization modality impact on MACE (HR 1.10, 95% CI .99–1.22, *p* = .07, *I* ^2^ = 23%).

We performed an additional sensitivity analysis according to the study design for the primary outcome. Pooled data from 3 randomized controlled trials showed that in male patients with ULMCA disease, PCI was associated with a higher risk of MACE compared with CABG (HR 1.30, 95% CI 1.10–1.53, *p*‐value .002, *I* ^2^ = 2%). Similarly, women with ULMCA disease who underwent PCI had a higher risk of MACE compared to CABG (HR 1.51, 95% CI 1.03–2.21, *p* value .04, *I* ^2^ = 33%). In contrast, the pooled data from 6 observational studies did not show any significant differences with regard to MACE in either men or women (men: HR 1.12, 95% CI .91–1.39, *p*‐value = .29; women: HR 1.17, 95% CI .77–1.79, *p* = .46) in patients undergoing either PCI or CABG. See Figures S5 and S6.

## 4 DISCUSSION

In this meta‐analysis of RCTs and adjusted observational studies, we compared outcomes after PCI or CABG for ULMCA disease according to patients' sex. The main findings can be summarized as follows:

1. Among male patients with ULMCA disease, PCI was associated with a higher risk of MACE compared to CABG.
2. The primary analysis showed that in female patients with ULMCA disease, PCI and CABG may be associated with a similar risk of MACE. However, such finding was driven by observational studies, and a moderator analysis did not find a significant interaction between sex and revascularization strategy for MACE.
3. There are no differences between the revascularization modalities in terms of all‐cause mortality, regardless of patients' sex.
4. PCI is associated with a significantly higher risk of repeated revascularization compared to CABG in both male and female patients, with a threefold increased risk in males and a fourfold increased risk in females.

Our meta‐analysis suggests the possibility to consider sex differences when choosing between the two revascularization strategies in patients with ULMCA disease. It is worth to be noted that, apart from the DELTA registry, the IRIS‐MAIN and the study by Hu et al.^10^, ^21^, ^23^ female patients in our study were allocated to receive PCI or CABG in a nearly 1:1 ratio.

The role of PCI and CABG in ULMCA revascularization has been examined in several previous meta‐analyses.^24^, ^25^, ^26^ Our study aims to build on this body of work by introducing novel insights, focusing specifically on the differential impact of revascularization strategies according to sex. Unlike prior meta‐analyses, which primarily assessed overall outcomes or included sex as a subgroup variable, we deliberately designed this study to evaluate sex as a central, predefined factor. By doing so, we investigated the potential interaction between sex and major outcomes such as major adverse cardiovascular events, all‐cause mortality, and repeated revascularization. Additionally, while previous meta‐analyses largely relied on RCTs, we included both RCTs and adjusted observational studies, thereby broadening the generalizability of our findings. This approach allowed us to confirm the consistency of prior findings regarding all‐cause mortality while generating hypothesis‐raising results for other endpoints, such as repeated revascularization and MACE, that may have sex‐specific implications in the PCI versus CABG debate. On the other hand, the inclusion of observational studies in this meta‐analysis, although adjusted with several statistical methods, contributed to the observed heterogeneity, particularly for the primary outcome. Despite conducting an additional sensitivity analysis to address this issue, the high heterogeneity remains a factor that warrants acknowledgment.

Overall, the significantly lower risk of MACE with CABG in males but not in females should be interpreted with caution. This finding was mainly driven by the results from the DELTA, the NOBLE and the PRECOMBAT trials.^10^, ^16^, ^17^ There is ongoing debate about whether sex should be taken into account when choosing the revascularization strategy, and prior studies evaluating outcomes based on sex have shown discordant findings.^27^, ^28^ Female patients experience higher mortality and morbidity in surgical settings compared to males.^29^, ^30^

Female sex has been reported as an independent predictor of worse postoperative outcomes, including mortality and morbidity after CABG.^31^ Furthermore, a higher incidence of postoperative complications such as MI, prolonged mechanical ventilation, hemodynamic support, deep sternal wound infections, postoperative sepsis and respiratory failure has been reported in female patients undergoing cardiac surgery.^32^

Consistently, several pre‐operative risk scoring systems, including the EuroSCORE^33^ and the STS (Society for Thoracic Surgeons Risk Modelling) score^34^ include female sex as one of the predictors for worse outcomes after surgical revascularization. Furthermore, when the SYNTAX trial demonstrated that female patients undergoing PCI had a higher adjusted 4‐year mortality risk compared to their male counterpart, sex became the major determinant in the SYNTAX Score II model that is currently used as a supporting tool to choose the best revascularization method^35^ in complex CAD. Such worse outcomes in female patients may also be associated with an older age at presentation, and it has been consistently demonstrated that advanced age is associated with poorer outcomes following cardiac surgery.^36^ Therefore, while the equipoise between PCI and CABG in terms of MACE in female patients shown in our meta‐analysis could be supported by a higher incidence of surgical complications in female sex in accordance with literature, this should be seen in the context of a limited sample compared to male patients. Further, it is important to acknowledge that our moderator analysis did not find a significant interaction between sex and revascularization strategy for MACE (*p* for interaction .422), indicating that the differences observed in our primary analysis may not reflect a true effect modification by sex. Additionally, the discordance in findings between observational studies and RCTs regarding the outcomes of PCI and CABG in women warrants further exploration.

While CABG is known to be associated with more favourable outcomes compared to PCI in the overall population, this difference has historically been attributed to the ability of CABG to reduce the rate of repeated revascularization over a long‐term follow‐up.^3^, ^37^ CABG, indeed, offers the advantage of overcoming the overall burden of atherosclerotic disease and achieving higher rates of complete revascularization as compared to PCI.^38^ PCI instead is usually restricted to flow‐limiting lesions and can be technically challenging in patients with diffuse CAD, often resulting in incomplete revascularization. In our analysis, such results were confirmed in both sexes, albeit the risk of repeated revascularization with PCI was higher in females as compared with males.

Although various factors are known to influence sex differences in patients with atherosclerotic CAD, such as hormonal effects and the prevalence of comorbidities,^39^ it is likely that anatomical differences and frailty also play a significant role. Female patients tend to have smaller vessel lumen diameters, more diffuse CAD and greater tortuosity, making them less suitable for percutaneous revascularization. This can potentially result in incomplete revascularization, higher risk of coronary stent late lumen loss and ultimately higher rate of target lesion revascularization with PCI.^40^ On the other side, it should be acknowledged that smaller coronary artery diameters and complex CAD may also pose challenges in the surgical setting, as this could lead to receiving less all‐arterial revascularization and overall less arterial grafting compared with male patients.^41^

A meta‐analysis of 6 studies assessing outcomes of female patients with multivessel or left main disease based on the revascularization strategy (CABG vs. PCI) concluded that PCI was associated with a higher incidence of adverse events, suggesting CABG as the preferred strategy in females. However, this study included both ULMCA disease and multivessel CAD.^42^ A more recent meta‐analysis of 6 studies including only patients with ULMCA disease concluded that PCI was associated with higher rates of MACE and MI in females compared to males, reinforcing the concept that CABG may be a better revascularization strategy for female patients with ULMCA disease.^43^ Taken together, existing evidence and our results could lead to re‐evaluate the approach to ULMCA disease in female patients based on comorbidities, modality of presentations, technical challenges and anatomical complexity.

Notably, the use of current antiplatelet therapies has reduced the risk of both MACE and all‐cause mortality in patients with CAD, regardless of patient's sex, even though an increased risk of bleeding is well established.^44^ Such additional concerns should be considered when deciding between PCI or CABG.

The fact that, as shown in our meta‐analysis, clinical outcomes following myocardial revascularization differ between sexes, that females have been underrepresented in RCTs enrolling both sexes, and that their representation in such studies has not changed over the last 20 years,^45^ supports the urgent need for new trials specifically designed for female only.

To this aim, the multicenter, randomized RECHARGE Trial – Women (RECHARGE:W – REvascularization CHoices Among Under‐Represented Groups Evaluation; NCT06399692) will assess both survival and quality‐of‐life in female patients in whom there is equipoise for revascularization with CABG and PCI. Moreover, the infrastructure of the ongoing ROMA Trial (Randomized Comparison of the Outcomes of Single vs. Multiple Arterial Grafts) will be leveraged to realize the first cardiac surgery trial dedicated to females (ROMA:Women; NCT04124120) to rigorously test the multiple arterial graft hypothesis in females.^46^

In consideration of the fact that most of the evidence on myocardial revascularization is currently derived from studies conducted in predominantly male populations and that generalization of the results to females may be inappropriate and potentially harmful, the results of these two RCTs are eagerly awaited not only to inform sex‐specific cardiovascular guidelines, but also to serve as potential models for future cardiovascular trialists.

### 4.1 Limitations

The findings of this meta‐analysis should be put in the context of several limitations. As with any meta‐analysis, our findings rely on aggregate data and share the limitations of each included study. Despite efforts to include RCTs and adjusted observational studies with a low risk of bias, the inferential power of our results may be constrained by the limited number of included studies and the paucity of data on outcomes such as all‐cause mortality and repeated revascularization; moreover, despite multivariate adjustment, observational studies might not have included in the analysis some prognostic variables that could account for the high heterogeneity observed for the primary endpoint. Additionally, there was heterogeneity in the definition of clinical endpoints across the studies. Our analysis included studies published between 2010 and 2023, and while this timeframe encompasses significant advancements in PCI and CABG techniques and antiplatelet regimens, it may not fully reflect contemporary patient cohorts. It should be acknowledged that, albeit included in the initial statistical plan, no sufficient data were retrieved to generate pooled estimates for secondary endpoints of interest such as stroke and myocardial infarction. Again, such a gap in evidence supports the need for future trials adequately powered to detect potential differences between males and females according to the chosen revascularization strategy.

## 5 CONCLUSIONS

In conclusion, among patients requiring coronary revascularization for ULMCA disease, CABG was associated with a lower risk of MACE compared with PCI in male but not in female patients. However, the equipoise between CABG and PCI in female patients was driven by observational studies, and a formal moderator analysis did not find a significant interaction between sex and revascularization strategy for the primary endpoint, suggesting caution in interpreting these results. CABG was confirmed to be associated with a lower rate of repeated revascularization compared to PCI, irrespective of patients' sex. No difference between PCI or CABG in terms of all‐cause mortality was observed in both sexes.

Our exploratory findings, although only hypothesis‐generating, suggest the possibility to account for sex differences when choosing between PCI and CABG in patients with ULMCA disease. Furthermore, they underscore the increasing need for balanced representation of sexes in future trials exploring this subject.

## AUTHOR CONTRIBUTIONS

All authors have contributed significantly to this work, participating in the conception, design, analysis, interpretation of data and writing/editing of this manuscript. O.D.F conceptualized and designed the study; M.D. performed the research; P.M., M.P., M.L.C., A.L., I.P., G.C., E.M. and S.S. collected and analysed the data; R.I. and G.D.P. analysed/interpreted the data and designed the figures; P.M., M.P., M.L.C., O.D.F., R.I. and G.D.P. drafted the article; F.B., F.D.A., A.C., G.M.D.F., M.G., M.M., A.D.F. and O.D.F critically revised the manuscript. The final manuscript was read and approved by all authors.

## CONFLICT OF INTEREST STATEMENT

Antonino Di Franco has consulted for Novo Nordisk, Servier and is an Advisory Board Member for Novo Nordisk and Scharper.

## Supporting information


Appendix S1.

Appendix S2.

## Data Availability

The data underlying this article are available in the article and in its online supplementary material. Any additional data will be shared on reasonable request to the corresponding author.
