# Supplementary material for: Percutaneous coronary intervention versus coronary artery bypass grafting in left main disease according to patients' sex: A meta‐analysis
Source: Eur J Clin Invest. 2024 Nov 14;55(2):e14348. doi: 10.1111/eci.14348 (PMC11744918; doi:10.1111/eci.14348)
Supplement: Supplementary file 2 — Appendix S2. [file ECI-55-e14348-s001.docx]

**Supplementary Material**

**Table of contents**

- Details of the full search strategy
- Supplementary Figure 1. RoB-2 for quality assessment of the randomized studies included in our analysis.
- Supplementary Figure 2. Funnel plot for MACE.
- Supplementary Figure 3. Moderator analysis.
- Supplementary Figure 4. Sensitivity analysis for MACE.
- Supplementary Figure 5. Subgroup analysis of MACE in males undergoing PCI vs CABG based on the design of the study
- Supplementary Figure 6. Subgroup analysis of MACE in females undergoing PCI vs CABG based on the design of the study.
- Supplementary Table 1. Inclusion and exclusion criteria.
- Supplementary Table 2. Baseline population characteristics.
- Supplementary Table 3. Procedural details (PCI).
- Supplementary Table 4. Procedural details (CABG).
- Supplementary Table 5. Newcastle-Ottawa Scale for quality assessment of the non-randomized studies included in our analysis.
- Supplementary Table 6. Outcomes definitions.

**Details of the full search strategy**

**Ovid MEDLINE** (ALL - 1946 to present)

Searched on January 31, 2024

No language, publication date, or article type restrictions

| **Line #** | **Search** |
| --- | --- |
|  | **Percutaneous Coronary Intervention/** |
|  | (percutaneous coronary intervention* or percutaneous coronary revasculari*ation* or percutaneous coronary angioplast* or PCI).tw. |
|  | **Stents/ or Drug-Eluting Stents/ or Self Expandable Metallic Stents/** |
|  | (stent or stents or stenting or stented).tw |
|  | **Angioplasty, Balloon, Coronary/** |
|  | (coronary balloon angioplast* or transluminal coronary balloon dilation or coronary artery balloon dilation or percutaneous transluminal coronary angioplast* or PTCA).tw. |
|  | **Atherectomy, Coronary/** |
|  | (coronary atherectom* or rotational atherectom*).tw |
|  | or/1-8 |
|  | **Coronary Artery Bypass/** |
|  | **Coronary Artery Bypass, Off-Pump/** |
|  | (coronary adj2 (bypass* or graft* or surger*)).tw. |
|  | (CABG or aorticocoronary anastomosis or total arterial revasculari*ation* or multiple arterial revasculari*ation*).tw. |
|  | **Internal Mammary-Coronary Artery Anastomosis/** |
|  | ((right internal mammary artery or RIMA or left internal mammary artery or LIMA or Coronary Internal Mammary Artery or arteria mammaria interna or arteria thoracica interna or internal thoracic artery or mammary internal artery) and (transplant* or graft* or anastomosis)).tw. |
|  | (surgical revasculari*ation* or cardiac muscle revasculari*ation* or coronary revasculari*ation* or heart muscle revasculari*ation* or heart myocardium revasculari*ation* or heart revasculari*ation* or internal mammary arterial anastomosis or internal mammary arterial implant* or internal mammary artery anastomosis or internal mammary artery graft* or internal mammary artery implant* or internal mammary-coronary artery anastomosis).tw. |
|  | **Myocardial Revascularization/** |
|  | (myocardial revasculari*ation* or myocardium revasculari*ation* or mammary artery implant* or mammary arterial implant* or mammary artery reimplant* or mammary arterial reimplant* or vineberg operation*).tw. |
|  | **Transmyocardial Laser Revascularization/** |
|  | (transmyocardial laser revasculari*ation* or trans-myocardial laser revasculari*ation*).tw. |
|  | or/10-20 |
|  | **Coronary Vessels/** and (left main).tw |
|  | ((left main) adj3 (artery or arteries or arterial or vessel or vessels or vein or veins)).tw |
|  | **Coronary Stenosis/** and (left main).tw |
|  | ((left) adj3 (stenosis or stem* or disease* or obstruction* or constriction* or stricture* or revasculari*ation*)).tw |
|  | or/22-25 |
|  | 9 and 21 and 26 |

**Ovid Embase** (1974-present)

Searched on January 31, 2024

No language, publication date, or article type restrictions

| **Line #** | **Search** |
| --- | --- |
|  | **percutaneous coronary intervention/** |
|  | (percutaneous coronary intervention* or percutaneous coronary revasculari*ation* or percutaneous coronary angioplast* or PCI).tw. |
|  | **stent/ or cardiovascular stent/ or drug eluting stent/ or metal stent/ or self expanding stent/** |
|  | (stent or stents or stenting or stented).tw |
|  | **transluminal coronary angioplasty/** |
|  | (coronary balloon angioplast* or transluminal coronary balloon dilation or coronary artery balloon dilation or percutaneous transluminal coronary angioplast* or PTCA).tw. |
|  | **coronary atherectomy** |
|  | (coronary atherectom* or rotational atherectom*).tw |
|  | or/1-8 |
|  | **coronary artery bypass graft/** |
|  | **off pump coronary artery bypass/** |
|  | (coronary adj2 (bypass* or graft* or surger*)).tw. |
|  | (CABG or aorticocoronary anastomosis or total arterial revasculari*ation* or multiple arterial revasculari*ation*).tw. |
|  | **heart muscle revascularization/** |
|  | ((right internal mammary artery or RIMA or left internal mammary artery or LIMA or Coronary Internal Mammary Artery or arteria mammaria interna or arteria thoracica interna or internal thoracic artery or mammary internal artery) and (transplant* or graft* or anastomosis)).tw. |
|  | (surgical revasculari*ation* or cardiac muscle revasculari*ation* or coronary revasculari*ation* or heart muscle revasculari*ation* or heart myocardium revasculari*ation* or heart revasculari*ation* or internal mammary arterial anastomosis or internal mammary arterial implant* or internal mammary artery anastomosis or internal mammary artery graft* or internal mammary artery implant* or internal mammary-coronary artery anastomosis).tw. |
|  | (myocardial revasculari*ation* or myocardium revasculari*ation* or mammary artery implant* or mammary arterial implant* or mammary artery reimplant* or mammary arterial reimplant* or vineberg operation*).tw. |
|  | (transmyocardial laser revasculari*ation* or trans-myocardial laser revasculari*ation*).tw. |
|  | or/10-18 |
|  | **coronary blood vessel/** and (left main).tw |
|  | ((left main) adj3 (artery or arteries or arterial or vessel or vessels or vein or veins)).tw |
|  | **left coronary artery/** |
|  | **coronary artery obstruction/** and (left main).tw |
|  | ((left) adj3 (stenosis or stem* or disease* or obstruction* or constriction* or stricture* or revasculari*ation*)).tw |
|  | or/20-24 |
|  | 9 and 19 and 25 |

**Cochrane Library** (Wiley)

Searched on January 31, 2024

| #1 MeSH descriptor: [Percutaneous Coronary Intervention] this term only  #2 percutaneous coronary intervention or percutaneous coronary revascularization or percutaneous coronary angioplast* or PCI  #3 MeSH descriptor: [Stents] explode all trees  #4 stent or stents or stenting or stented  #5 MeSH descriptor: [Angioplasty, Balloon, Coronary] this term only  #6 coronary balloon angioplast* or transluminal coronary balloon dilation or coronary artery balloon dilation or percutaneous transluminal coronary angioplast* or PTCA  #7 MeSH descriptor: [Atherectomy, Coronary] this term only  #8 #1 or #2 or #3 or #4 or #5 or #6 or #7  #9 MeSH descriptor: [Coronary Artery Bypass] 1 tree(s) exploded  #10 coronary NEAR/2 (bypass* or graft* or surger*)  #11 CABG  #12 aorticocoronary anastomosis or total arterial revascularization or multiple arterial revascularization  #13 (right internal mammary artery or RIMA or left internal mammary artery or LIMA or Coronary Internal Mammary Artery or arteria mammaria interna or arteria thoracica interna or internal thoracic artery or mammary internal artery) and (transplant or graft or anastomosis) 598  #14 surgical revascularization or cardiac muscle revascularization or coronary revascularization or heart muscle revascularization or heart myocardium revascularization or heart revascularization  or internal mammary arterial anastomosis or internal mammary arterial implant* or internal mammary artery anastomosis or internal mammary artery graft* or internal mammary artery implant* or internal mammary-coronary artery anastomosis 768  #15 myocardial revascularization or myocardium revascularization or mammary artery implant* or mammary arterial implant* or mammary artery reimplant* or mammary arterial reimplant* or vineberg operation  #16 transmyocardial laser revascularization or trans-myocardial laser revascularization  #17 #9 or #10 or #11 or #12 or #13 or #14 or #15 or #16  #18 (left main) NEAR/3 (artery or arteries or arterial or vessel or vessels or vein or veins)  #19 (left) NEAR/3 (stenosis or stem* or disease* or obstruction* or constriction* or stricture*)  #20 #18 or #19  #21 #8 and #17 and #20 |
| --- |

**Supplementary Figure 1.** RoB-2 for quality assessment of the randomized studies included in our analysis.


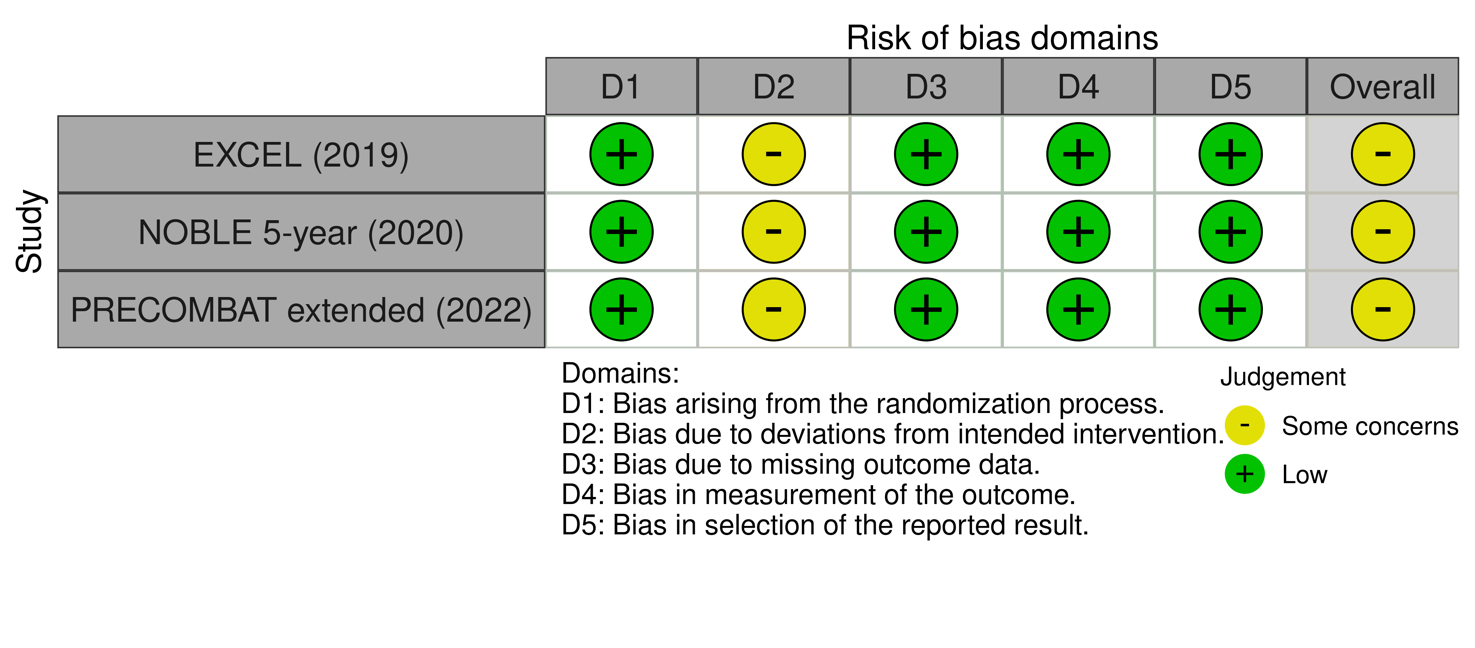


**Supplementary Figure 2.** Funnel plot for major adverse cardiovascular events (MACE) in females (left panel) and males (right panel).


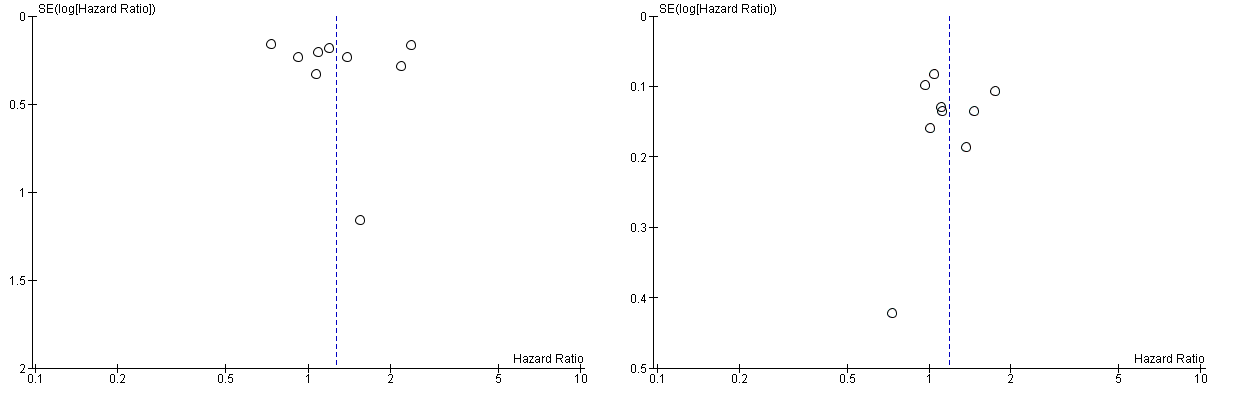


SE, Standard Error.

**Supplementary Figure 3**. Moderator analysis ('sex' as moderator) on primary outcome.


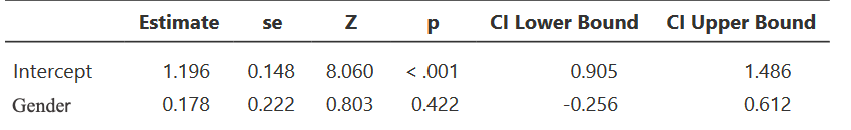


**Supplementary Figure 4.** Sensitivity analysis for the primary endpoint to lower heterogeneity.

**
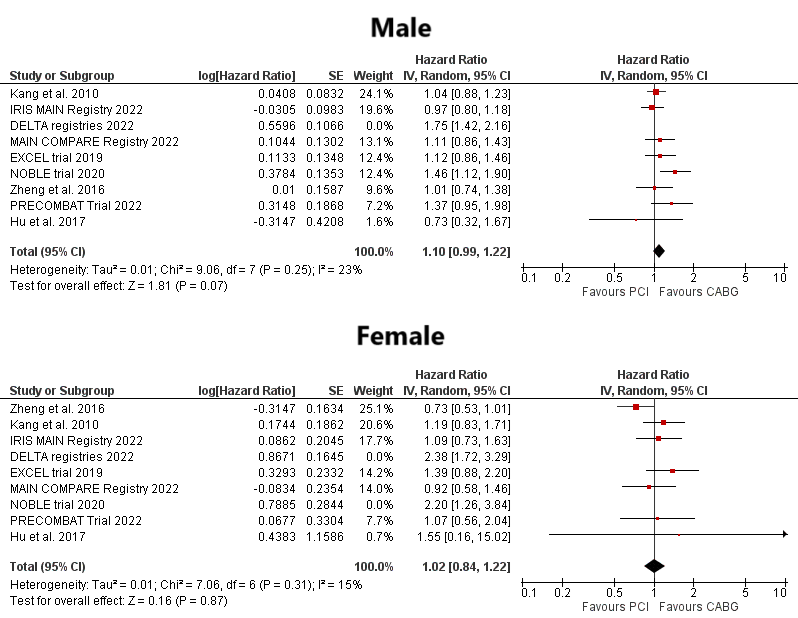
**

**Supplementary Figure 5**. Subgroup analysis of MACE in males undergoing PCI vs CABG based on the design of the study


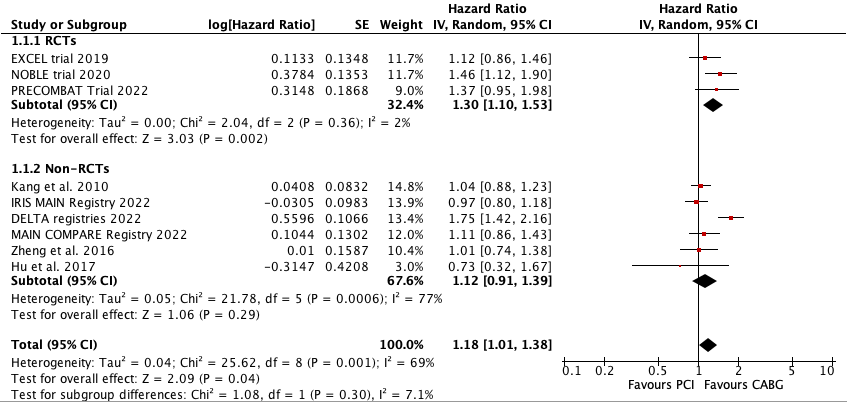


**Supplementary Figure 6**. Subgroup analysis of MACE in females undergoing PCI vs CABG based on the design of the study.


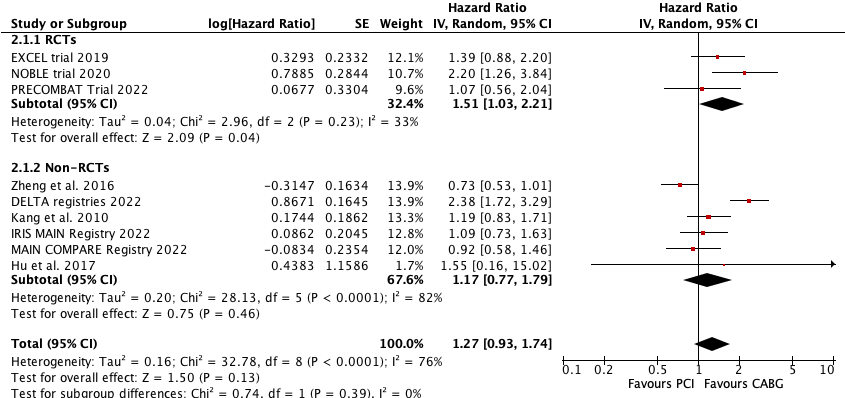


**Supplementary table 1**. Inclusion and exclusion criteria of included studies.

| **STUDY** | **Inclusion criteria** | **Exclusion criteria** |
| --- | --- | --- |
| **Kang et al., 2010^18^** | Patients with unprotected LMCA (≥ 50% stenosis) or LMCA-equivalent disease (≥ 70% proximal LAD and proximal LCX stenoses) treated with CABG or PCI with DES, including those who presented with acute MI or cardiogenic shock and those who underwent emergency procedures | Patients not meeting inclusion criteria |
| **Zheng et al., 2016^19^** | Patients with angiographically confirmed ULMD (LMCA luminal narrowing > 50% without patent bypass grafts to its branches) and treated with PCI or CABG | Patients younger than 18 years of age, with prior CABG, concomitant valvular, or aortic surgery, ST-segment elevation myocardial infarction within 1 week or cardiogenic shock |
| **Hu et. al, 2017^23^** | “All comers” patients with ULMCA true bifurcation lesions (≥ 50% diameter stenosis) diagnosed by angiography treated with either PCI with DES or CABG | Patients not meeting inclusion criteria |
| **EXCEL, 2019^15^** | All of the following must be present:  1A. ULMCA disease with angiographic diameter stenosis ≥70% (visually estimated), or with angiographic diameter stenosis ≥50% but <70% with one or more of the following present: a) Non-invasive evidence of ischaemia referable to a haemodynamically significant LM lesion, and/or b) Intravascular Ultrasound (IVUS) minimal lumen area (MLA) ≤6.0 mm2, and/or c) FFR ≤0.80  OR  1B. LM equivalent disease: LM distal bifurcation (Medina 0,1,1) disease, in the absence of significant angiographic stenosis in the LM coronary artery, may also be randomised if either of the following conditions is present: a) Both the ostial LAD and ostial LCX stenoses are ≥70% stenotic by visual estimation, or b) If one or both of the ostial LAD and ostial LCX stenoses are ≥50% and <70% by visual estimation, then this lesion(s) is demonstrated to be significant either by non-invasive evidence of ischaemia in its myocardial distribution; and/or FFR ≤0.80; and/or IVUS MLA ≤4.0 mm2.  2. Clinical and anatomic eligibility for both PCI and CABG as agreed to by the local Heart Team (interventionalist determines PCI appropriateness and eligibility; cardiac surgeon determines surgical appropriateness and eligibility)  3. ≥18 years of age  4. Ability to sign informed consent and comply with all study procedures, including follow-up for at least three years | All of the following must be absent:  1. Prior PCI of the LM trunk at any time prior to randomisation, PCI of any other (non-LM) coronary artery lesions within one year prior to randomisation, and CABG at any time prior to randomisation  2. Need for any concomitant cardiac surgery other than CABG (e.g., valve surgery, aortic repair, etc.), or intent that, if the subject randomises to surgery, any cardiac surgical procedure other than isolated CABG will be performed  3. The presence of any clinical condition(s) which leads the participating interventional cardiologist to believe that clinical equipoise is not present (i.e., the subject should not be treated by PCI, but rather should be managed with CABG or medical therapy)  4. The presence of any clinical condition(s) which leads the participating cardiac surgeon to believe that clinical equipoise is not present (i.e., the subject should not be treated by CABG, but rather should be managed with PCI or medical therapy)  5. Subjects unable to tolerate, obtain or comply with dual antiplatelet therapy for at least one year  6. Subjects requiring or who may require additional surgery (cardiac or non-cardiac) within one year  7. CK-MB greater than the local laboratory upper limit of normal, or recent MI with CK-MB levels still elevated  8. Pregnancy or intention to become pregnant  9. Non-cardiac comorbidities with life expectancy <3 years  10. Current participation in other investigational drug or device studies that have not reached their primary endpoint  11. Angiographic exclusion criteria: a) LM diameter stenosis <50%, unless LM equivalent disease is present, b) SYNTAX score ≥33, as determined by the local Heart Team, c) Visually estimated LM reference vessel diameter <2.25 mm or >4.25 mm, d) The presence of specific coronary lesion characteristics or other cardiac condition(s) which leads the participating interventional cardiologist or cardiac surgeon to believe that clinical equipoise is not present |
| **NOBLE 5-year, 2020^17^** | - Stable angina pectoris, unstable angina pectoris, or acute coronary syndrome  - Significant lesion (visually assessed diameter of stenosis of >50% or FFR of ≤ 0,80) in the LM coronary artery ostium, mid-shaft, or bifurcation  - No more than three additional non-complex lesions (complex lesions defined as chronic total occlusions, bifurcation lesions requiring two stent techniques or lesions with calcified or tortuous  vessel morphology)  - Ability to provide informed consent | - ST-elevation myocardial infarction within 24 h  - Being considered at too high risk for CABG or PCI  - Expected survival of less than 1 year |
| **CREDO-Kyoto PCI/CABG Registry Cohort-3, 2021** | Patients who underwent first coronary revascularization with PCI or isolated CABG without combined non-coronary surgery | - Patients who refused study participation  - Patients with acute myocardial infarction  - Patients without ULMCAD |
| **DELTA registries, 2022^20^** | DELTA registry included all-comer patients with ULMCA disease treated in 14 centers with either PCI with first-generation DES or CABG between April 2002 and April 2006.  DELTA 2 registry included all-comer patients with ULMCA disease treated with PCI with new-generation DES in 19 centers in 7 countries between April 2006 and December 2015. | Patients not meeting inclusion criteria |
| **IRIS MAIN registry, 2022^21^** | “All-comer” patients with unprotected LMCA disease treated with medical therapy, PCI, or CABG from 50 academic and community hospitals in Asia (China, India, Indonesia, Japan, Malaysia, South Korea, Taiwan, and Thailand) from January 2003 and December 2016. | - Prior cardiac surgery  - Concomitant valvular or aortic surgery  - Patients who received medical treatment alone  - Unavailable information on the coronary anatomy |
| **PRECOMBAT Extended, 2022^16^** | 1. Patient must be at least 18 years of age.  2. Patient must have significant de novo ULMCA stenosis (>50% by visual estimation) with or without any additional target lesions (>70% by visual estimation). “Unprotected” in this context means that no perfusion distal to the LM stenosis is supplied by either a patent bypass graft or a collateral vessel.  3. LM lesions and lesions outside ULMCA (if present) must be potentially comparably treatable with PCI and CABG  4. Patients with stable (CCS class 1 to 4) or acute coronary syndromes (unstable angina pectoris Braunwald class IB, IC, IIB, IIC, IIIB, IIIC or Non-ST elevation MI) or patients with atypical chest pain or without symptoms but having documented myocardial ischemia may be enrolled.  5. Patient or guardian must agree to the study protocol and the schedule of clinical and angiographic follow-up, and must provide informed, written consent, as approved by the appropriate Institutional Review Board/Ethical Committee of the respective clinical site. | 1. Known hypersensitivity or contraindication to any of the following medications: Heparin, Aspirin, Both clopidogrel and ticlopidine, Sirolimus, Stainless steel and/or, Contrast media (patients with documented sensitivity to contrast which can be effectively pre-medicated with steroids and diphenhydramine [e.g. rash] may be enrolled. Patients with true anaphylaxis to prior contrast media, however, should not be enrolled).  2. Systemic (intravenous) sirolimus use within 12 months.  3. Any previous PCI within 1 year  4. Previous CABG  5. Any previous PCI of a ULMCA or ostial LCX artery or ostial LAD artery lesion within 1 year  6. Intention to treat more than one totally occluded major epicardial vessel  7. Acute MI within 1 week  8. Ejection fraction <30%.  9. Cardiogenic shock  10. Any stroke with a persistent neurological deficit or any cerebrovascular accident within 6 months  11. Creatinine level ≥ 2.0mg/dL or dependence on dialysis.  12. Severe hepatic dysfunction (AST and ALT ≥ 3 times upper normal reference values).  13. Gastrointestinal or genitourinary bleeding within the prior 3 months, or major surgery within 2 months.  14. History of bleeding diathesis or known coagulopathy (including heparin-induced thrombocytopenia) or will refuse blood transfusions.  15. Current known current platelet count <100,000 cells/mm3 or haemoglobin <10 g/dL.  16. A planned elective surgical procedure that would necessitate interruption of thienopyridines during the first 1-year post enrolment.  17. Non-cardiac co-morbid conditions with life expectancy <1 year or that may result in protocol non-compliance (per site investigator’s medical judgment).  18. Active participation in another drug or device investigational study, which has not completed the primary end point follow-up period.  19 Unable or unwilling to follow-up with visits required by protocol  20. Female of childbearing potential, unless a recent pregnancy test is negative, who possibly plan to pregnant any time after enrolment into this study |
| **MAIN COMPARE Extended, 2023^22^** | Consecutive patients with significant LMCA disease who underwent PCI or CABG in 12 major centers in Korea between January 2000 and June 2006 | Patients with previous CABG, concomitant valve or aortic surgery, or ST-segment–elevation MI or cardiogenic shock at presentation |

CABG, coronary artery bypass grafting; CK-MB, creatine kinase-myocardial band; DES, drug-eluting stent; FFR, Fractional Flow Reserve; LAD, left anterior descending; LCX, left circumflex; LMCA, left main; LMCAD, left main coronary artery disease; MI, myocardial infarction; ULMCA, unprotected left main coronary artery; ULMD, unprotected left main disease.

**Supplementary Table 2.** Baseline populations characteristics.

| **Study** | **Number of patients** | | | | **HTN (%)** | **DM (%)** | **Smokers (%)** | **Dyslipidemia (%)** | **Previous PCI (%)** | **Prior stroke (%)** | **LVEF (mean)** | **CKD (%)** | **ACS (%)** | **STEMI (%)** | **NSTEMI (%)** | **Unstable Angina (%)** | **Stable CAD (%)** |
| --- | --- | --- | --- | --- | --- | --- | --- | --- | --- | --- | --- | --- | --- | --- | --- | --- | --- |
|  | **Males, PCI** | **Males, CABG** | **Females, PCI** | **Females, CABG** |  |  |  |  |  |  |  |  |  |  |  |  |  |
| Kang et al., 2010^18^ | 144 | 190 | 61 | 67 | 66 | 41 | 47 | 57 | 12 | 12 | 55 | 11 | 64 | N/A | N/A | 48 | 36 |
| Zheng et al., 2016^19^ | 1134 | 2135 | 308 | 469 | 61 | 29 | 51 | 56 | 14 | N/A | 61 | N/A | 52 | N/A | N/A | 52 | 48 |
| Hu et. al, 2017^23^ | 180 | 56 | 28 | 12 | 64 | 34 | 50 | 45 | 19 | 6 | 59 | N/A | 89 | 9 | 10 | 70 | 11 |
| EXCEL, 2019^15^ | 722 | 742 | 226 | 215 | 74 | 29 | 22 | 70 | 17 | 6 | 57 | 16 | 39 | 1 | 13 | 25 | 61 |
| NOBLE 5-year, 2020^17^ | 476 | 452 | 116 | 140 | 65 | 15 | 20 | 80 | 20 | N/A | 60 | N/A | 17 | N/A | N/A | N/A | 83 |
| CREDO-Kyoto PCI/CABG Registry Cohort-3, 2021^20^ | 288 | 376 | 95 | 96 | 82 | 46 | 16 | 67 | 0 | 17 | 61 | 11 | 4 | 0 | 0 | 4 | 96 |
| DELTA registries, 2022^10^ | 4002 | 562 | 1365 | 324 | 73 | 31 | 40 | 68 | 33 | N/A | 53 | 20 | 18 | 5 | N/A | N/A | 82 |
| IRIS MAIN registry, 2022^21^ | 2214 | 1138 | 650 | 318 | 63 | 37 | 25 | 61 | 16 | 8 | 58 | 4 | 60 | 5 | 10 | 45 | 40 |
| PRECOMBAT Extended, 2022^16^ | 228 | 231 | 72 | 69 | 53 | 32 | 29 | 41 | 13 | N/A | 60 | 1 | 50 | N/A | N/A | 45 | 50 |
| MAIN COMPARE Extended, 2023^22^ | 779 | 830 | 323 | 308 | 49 | 32 | 28 | 31 | 15 | 7 | 59 | 3 | 72 | 0 | 10 | 62 | 28 |

ACS, acute coronary syndrome; CABG, coronary artery by-pass grafting; CAD, coronary artery disease; CKD, chronic kidney disease; DM, diabetes mellitus; HTN, hypertension; LVEF, left ventricular ejection fraction; N/A, not available/applicable; NSTEMI, non ST-elevated myocardial infarction; PCI, percutaneous coronary intervention; STEMI, ST-elevated myocardial infarction

**Supplementary Table 3.** Procedural details (PCI).

| **Study** | **Number of stents** | **Single stent (%)** | **Culotte (%)** | **Crushing technique (%)** | **Kissing (%)** | **T stent technique (%)** | **Drug** | **Intravascular Imaging** |
| --- | --- | --- | --- | --- | --- | --- | --- | --- |
| Kang et al., 2010^18^ | 1.37 | 65.9 | N/A | 9.3 | 14.1 | 10.7 | Sirolimus (70.2%) Paclitaxel (26.3%) Zotarolimus (3.4%) | N/A |
| Zheng et al., 2016^19^ | 2.2 | 72.3 | 1.5 | 18.8 | 3.6 | 3.8 | N/A | 38.80% (IVUS) |
| Hu et. al, 2017^23^ | 1.47 | 46.2 | 24.5 | 7.7 | N/A | 16.8 | N/A | 30.30% (IVUS) |
| EXCEL, 2019^15^ | 2.4 | N/A | N/A | N/A | N/A | N/A | Everolimus (99.2%) | 77.20% (IVUS) |
| NOBLE 5-year, 2020^17^ | N/A | 65.0 | 23.9 | 4.0 | N/A | 8.4 | N/A | 75.00% (IVUS) |
| CREDO-Kyoto PCI/CABG Registry Cohort-3, 2021^20^ | 2 | N/A | N/A | N/A | N/A | N/A | Everolimus (65.8%) Biolimus (46.9%) Zotarolimus (8.6%) | 92.40% (IVUS) 92.20% (OCT) |
| DELTA registries, 2022^10^ | N/A | N/A | N/A | N/A | N/A | N/A | N/A | 38.00% (IVUS) |
| IRIS MAIN registry, 2022^21^ | 2.28 | N/A | N/A | N/A | N/A | N/A | N/A | 78.00% (IVUS) |
| PRECOMBAT Extended, 2022^16^ | 2 | N/A | N/A | N/A | N/A | N/A | Sirolimus (100%) | N/A |
| MAIN COMPARE Extended, 2023^22^ | 1.2 | 83.9 | N/A | N/A | N/A | N/A | Sirolimus (55.4%) Paclitaxel (15.6%) | 74.80% (IVUS) |

IVUS, intravascular ultrasound; N/A, not available/applicable; OCT, optical coherence tomography.

**Supplementary Table 4.** Procedural details (CABG).

| **Study** | **Total graft (N)** | **Arterial graft (%)** | **Vein graft (%)** | **Use of LIMA (%)** | **Use of IMA (%)** | **Off-pump surgery (%)** |
| --- | --- | --- | --- | --- | --- | --- |
| Kang et al., 2010^18^ | 3.2 | ≥ 94.0 | 29.6 | 94.2 | ≥ 94.0 | 71.6 |
| Zheng et al., 2016^19^ | 3.4 | N/A | N/A | N/A | 94.2 | 53.3 |
| Hu et. al, 2017^23^ | 2.5 | N/A | N/A | N/A | N/A | 61.8 |
| EXCEL, 2019^15^ | 2.6 | 98.4 | N/A | N/A | 98.8 | 29.4 |
| NOBLE 5-year, 2020^17^ | 2.5 | ≥ 96.0 | 5.0 | N/A | N/A | 15.6 |
| CREDO-Kyoto PCI/CABG Registry Cohort-3, 2021^20^ | N/A | N/A | N/A | N/A | 97.5 | 58.3 |
| DELTA registries, 2022^10^ | N/A | N/A | N/A | N/A | N/A | N/A |
| IRIS MAIN registry, 2022^21^ | 3.1 | N/A | N/A | 94.0 | N/A | 57.0 |
| PRECOMBAT Extended, 2022^16^ | 3.5 | 93.4 | N/A | 93.0 | N/A | 60.2 |
| MAIN COMPARE Extended, 2023^22^ | 2.8 | 96.9 | N/A | 96.9 | N/A | 41.7 |
|  |  |  |  |  |  |  |

LIMA, left internal mammary artery; IMA, internal mammary artery; N/A, not available/applicable.

**Supplementary Table 5.** Newcastle-Ottawa Scale for quality assessment of the non-randomized studies included in our analysis.

| **Study** | **Selection** | **Comparability** | **Outcome** |
| --- | --- | --- | --- |
| **Kang et al., 2010**^18^ | ******** | ****** | ****** |
| **Zheng et al., 2016**^19^ | ******** | ****** | ****** |
| **Hu et al., 2023**^23^ | ******** | ****** | ***** |
| **CREDO-Kyoto PCI/CABG Registry Cohort-3, 2021**^20^ | ******** | ****** | ****** |
| **DELTA registry, 2022**^10^ | ******** | ****** | ****** |
| **IRIS-MAIN registry, 2017**^21^ | ******** | ****** | ****** |
| **MAIN COMPARE Extended, 2023**^22^ | ******** | ****** | ******* |

Good quality: 3 or 4 asterisks in selection domain AND 1 or 2 asterisks in comparability domain AND 2 or 3 asterisks in outcome/exposure domain. Fair quality: 2 asterisks in selection domain AND 1 or 2 asterisks in comparability domain AND 2 or 3 asterisks in outcome/exposure domain. Poor quality: 0 or 1 asterisk in selection domain OR 0 asterisks in comparability domain OR 0 or 1 asterisk in outcome/exposure domain.

**Supplementary table 6**. Outcomes definitions.

| **STUDY** | **MACE** | **All-Cause Death** | **Repeat Revascularization** |
| --- | --- | --- | --- |
| **Kang et al., 2010**^18^ | Composite of death, MI, CVA, or TVR. | Death was classified as from either cardiac or non-cardiac causes, according to the ARC definition. All deaths were considered cardiac in origin unless a noncardiac origin had definitely been documented. | TVR was defined as the repeat intervention (surgical or percutaneous) of any segment of the treated vessel, including the LM, LAD, and LCX |
| **Zheng et al., 2016**^19^ | Composite of death, nonfatal MI, or nonfatal stroke. | Death from any cause | Repeat revascularization was defined as any repeat PCI or CABG. All stages of a staged index PCI procedure will be considered part of the index revascularization procedure and not a repeated revascularization. |
| **Hu et. al, 2017**^23^ | Composite of all-cause death, MI, stroke or TVR | Death was defined as postprocedure death from any cause and classified as from either cardiac or noncardiac causes, according to the ARC definition. | TVR was defined as any surgical or percutaneous repeat revascularization of any segment of the stented vessel (target lesion, upstream or downstream branches) within 1-year, including the LM, LAD and LCX coronary arteries. A planned staged PCI was not considered as a TVR. |
| **EXCEL, 2019**^15^ | Composite of all-cause death, MI, or stroke | Death from cardiovascular causes, non-cardiovascular causes, or undetermined causes.  Non-cardiovascular death is defined as any death with known cause not of cardiac or vascular cause | A coronary revascularisation procedure may be either a CABG or a PCI. The coronary segments revascularised will be sub-classified as: • TLR: a lesion revascularised in the index procedure (or during a planned or provisional staged procedure). The LM target lesion extends from the LM stem ostium to the end of the 5 mm proximal segments of the LAD and LCX arteries as well as the ramus intermedius if the latter vessel has a vessel diameter of ≥2 mm. • TVR: the target vessel is defined as the entire major coronary vessel proximal and distal to the target lesion including upstream and downstream branches and the target lesion itself. The LM and any vessel originating from the LM coronary artery or its major branches is, by definition, considered a target vessel for the purposes of this trial (unless either the LAD or LCX is occluded at baseline and no attempt was made to revascularise these territories by either PCI or CABG). • Target vessel non-target lesion: the target vessel non-target lesion consists of a lesion in the epicardial vessel/branch/graft that contains the target lesion; however, this lesion is outside of the target lesion by at least 5 mm distal or proximal to the target lesion determined by quantitative coronary angiography (QCA). • Non-target vessel: for the purposes of this trial, the only possible non-target vessel would be the right coronary artery and its major branches that were not treated by either PCI or CABG at the index procedure (unless either the LAD or LCX is occluded at baseline and no attempt was made to revascularise these territories by either PCI or CABG).  Revascularisation will be considered ischaemia-driven if the diameter stenosis of the revascularised coronary segment is ≥50% by QCA and any of the following criteria for ischaemia are met: a) A positive functional study corresponding to the area served by the target lesion; or b) Ischaemic ECG changes at rest in a distribution consistent with the target vessel; or c) Typical ischaemic symptoms referable to the target lesion; or d) IVUS (Intravascular Ultrasound) of the target lesion with a minimal lumen area (MLA) of ≤4 mm2 for non-LM lesions or ≤6 mm2 for LM lesions. If the lesions are de novo (i.e., not restenotic), the plaque burden must also be ≥60%; or e) FFR (Fractional Flow Reserve) of the target lesion ≤0.80  A target lesion revascularisation for a diameter stenosis less than 50% might also be considered ischaemia-driven if there was a markedly positive functional study or ECG changes corresponding to the area served by the target lesion. |
| **NOBLE 5-year, 2020**^17^ | Composite of all-cause mortality, non-procedural myocardial infarction, repeat revascularisation, or stroke. | Death from any cause | Repeat revascularization was defined as any new PCI or CABG operation performed during follow–up. If an index revascularisation was attempted or successful, any subsequent revascularisation was counted as repeat revascularisation. TLR was defined as repeat revascularisation by PCI of any target segment treated during the index procedure. LM coronary artery revascularisation was defined as any subsequent revascularisation by PCI of the segments within 5 mm of any treated segment related to the LM or the LM bifurcation. Any revascularisation by CABG of native LM including the LM bifurcation, or revascularisation of a graft supplying the LAD or LCX arteries. |
| **CREDO-Kyoto PCI/CABG Registry Cohort-3, 2021**^20^ | N/A | Death from any cause. | TVR was defined as either PCI or CABG due to restenosis or thrombosis of the target vessel. LM-related revascularization was defined as either PCI or CABG for LM or for its side branch (LAD and LCX artery) ostial lesions. Any coronary revascularization was defined as either PCI or CABG for any reason. Ischemia-driven TVR, ischemia-driven LM-related revascularization, and ischemia-driven any coronary revascularization were adjudicated according to the ARC definition. |
| **DELTA registries, 2022**^10^ | Composite of all-cause death, MI, CVA, and TVR | Death from any cause. | TLR was defined as any repeat intervention of the target lesion or other complication of the target lesion. The target lesion was defined as the treated segment 5 mm proximally to the stent and 5 mm distally to the stent. TVR was defined as any repeat intervention of any segment of the target vessel, defined as the entire major coronary vessel proximal and distal to the target lesion, including upstream and downstream branches and the target lesion itself. |
| **IRIS MAIN registry, 2022**^21^ | Composite of death from any causes, MI, or stroke. | Death from any cause. | Repeat revascularization included any type of percutaneous or surgical revascularization procedure, regardless of whether the procedure was performed on a target or non-target lesion. |
| **PRECOMBAT Extended, 2022**^16^ | Composite of death, nonfatal MI, nonfatal stroke, or ischemia-driven TVR | Death from any cause. | TVR was defined as PCI or CABG in the treated vessel that was driven by ischemia (ie. stenosis of at least 50% the diameter of the target vessel with ischemic signs or symptoms or if the stenosis was at least 70% of the diameter of the target vessel regardless of ischemic signs or symptoms) |
| **MAIN COMPARE Extended, 2023**^22^ | Composite of death, Q-wave MI, or stroke | Death from any cause | TVR was defined as any repeat revascularization of the target vessels including any segments in LM, the LAD, and/ or LCX, 10 years after index revascularization |

ARC, Academic Research Consortium; CABG, coronary artery by-pass grafting; CVA, cerebrovascular accident; PCI, percutaneous coronary intervention; LAD, left anterior descending; LCX, left circumflex; LM, left main; MI, myocardial infarction; N/A, not applicable/available; TLR, target lesion revascularization; TVR, target vessel revascularization; ULN, upper limit of normal.
